# A Synthetic Time-Series Generation Using a Variational Recurrent Autoencoder with an Attention Mechanism in an Industrial Control System

**DOI:** 10.3390/s24010128

**Published:** 2023-12-26

**Authors:** Seungho Jeon, Jung Taek Seo

**Affiliations:** 1 Department of Computer Engineering (Smart Security), Gachon University, Seongnam-si 1342, Republic of Korea; shjeon90@gachon.ac.kr; 2 Department of Computer Engineering, Gachon University, Seongnam-si 1342, Republic of Korea

**Keywords:** synthetic data generation, time-series data, variational recurrent autoencoder, attention mechanism, industrial control system

## Abstract

Data scarcity is a significant obstacle for modern data science and artificial intelligence research communities. The fact that abundant data are a key element of a powerful prediction model is well known through various past studies. However, industrial control systems (ICS) are operated in a closed environment due to security and privacy issues, so collected data are generally not disclosed. In this environment, synthetic data generation can be a good alternative. However, ICS datasets have time-series characteristics and include features with short- and long-term temporal dependencies. In this paper, we propose the attention-based variational recurrent autoencoder (AVRAE) for generating time-series ICS data. We first extend the evidence lower bound of the variational inference to time-series data. Then, a recurrent neural-network-based autoencoder is designed to take this as the objective. AVRAE employs the attention mechanism to effectively learn the long-term and short-term temporal dependencies ICS data implies. Finally, we present an algorithm for generating synthetic ICS time-series data using learned AVRAE. In a comprehensive evaluation using the ICS dataset HAI and various performance indicators, AVRAE successfully generated visually and statistically plausible synthetic ICS data.

## 1. Introduction

Data scarcity is a significant obstacle for modern data science and artificial intelligence research communities. The fact that abundant data are a key element of powerful prediction models has now become generally recognized through transformer [1] and generative pre-training [2,3]. However, publishing high-quality datasets is very laborious and time-consuming. Even if data are collected, they are often difficult to disclose. Unfortunately, this is especially true for data from industrial control systems (ICS). Industrial control systems are computerized systems for operating and controlling industrial facilities and infrastructure, and are an essential element of manufacturing automation. Researchers put a lot of effort into acquiring ICS datasets to understand the dynamics of ICS or improve security. However, in general, only a few samples of the ICS dataset are released, or access from outside is restricted for organizational security reasons. Additionally, it is difficult to develop a robust prediction model due to data scale issues. In this environment, synthetic data generation is an excellent means to secure the diversity of datasets.

Dataset generation is one of the most important research topics in modern artificial intelligence research based on deep learning. The variational autoencoder (VAE) [4] statistically learns the representation of the latent variables and provides a powerful data generation method based on statistics. The generative adversarial network (GAN) [5] is currently one of the most powerful generative models and presents a framework for competitively learning the generator and the discriminator. VAE and GAN have different approaches to data generation, and several derivative models have been proposed since these models were introduced. Z. Wan et al. [6] proposed a VAE-based data generation algorithm for imbalanced learning. TimeGAN [7] presented a novel GAN-based architecture and learning algorithm for generating time-series data. CTGAN [8] uses the conditional generator to model continuous data included in the tabular data.

Unfortunately, generating synthetic ICS datasets has several challenges. First, in most cases, data collected in an ICS environment have time-series characteristics. In comparison, many synthetic data generation studies [9,10,11] are not suitable for time-series data generation because they assume the independently and identically distributed (i.i.d) data. Second, the ICS environment includes various devices like sensors, and the data collected here are naturally multivariate. Therefore, the generative model should be able to learn the dynamics of multiple devices jointly. Lastly, the time-series multivariate data like this include both short- and long-term temporal dependencies. For example, because equipment such as boilers should maintain a constant temperature, the data collected also have long-term patterns. In comparison, devices such as flow sensors often have relatively short-term patterns because they detect information about the environment in real time. These complex data characteristics make it difficult to learn generative models.

Our insights to overcome the above challenges were largely three-fold. First, we employed the recurrent neural network (RNN) to generate time-series data. RNN is a type of neural network with an internal cyclic structure to process patterns in sequential data. RNN has been successfully applied to various applications such as natural language processing [12,13], speech recognition [14,15], or video analysis [16,17]. However, RNN exhibits some problems, such as long-term dependency problems and a vanishing gradient as the length of the sequence increases. The long short-term memory (LSTM) [18] and the gated recurrent unit (GRU) [19] have solved some of these problems. However, due to the structural nature of processing data sequentially, past data are hard to maintain in the present or future. Our second insight, the attention mechanism, was employed to handle this issue. The attention mechanism allowed the model to utilize information effectively by paying attention to important parts of the given data when performing prediction tasks [20,21]. Third, we adopted the variational method to learn the generative model. The variational method is an approach adopted in VAE and an optimization technique for approximating complex probability distributions or functions. We approximated the ICS data’s distribution through the variational method. Most of the recent generative models have been designed based on GAN, but the learning progress of GAN is extremely unstable due to its nature. In comparison, the variational method enables more stable learning progress by learning the probability distribution of the data’s latent representation.

In this paper, we combined the above insights to propose the attention-based variational recurrent autoencoder (AVRAE) to generate time-series ICS data. AVRAE was designed with a similar structure to the variational recurrent autoencoder (VRAE) [22] proposed in 2015. This model was trained to maximize the evidence lower bound of the variational inference. However, the basic form of the evidence lower bound did not assume the sequential data. Therefore, we first extended the evidence lower bound to the sequential data. AVRAE basically had the same encoder and the decoder structure as the sequence-to-sequence (Seq2Seq) [23] mapping method. The encoder processed the input sequence to generate the latent vectors, and the decoder generated the same output as the input sequence from these latent vectors. While VRAE successfully mapped the sequential data to the latent space, it still failed to capture the complex dynamics of time-series data. We combined VRAE with the attention mechanism to calculate the relative importance between input sequences, allowing the model to reconstruct or generate sequences effectively. AVRAE’s attention layers were inspired by the transformer’s scaled-dot attention [1]. In other words, the attention weights were calculated using the hidden state at each timestep produced by AVRAE’s encoder and the hidden states produced by the decoder, and these weights multiplied by the hidden states of the encoder to become the output of AVRAE. AVRAE successfully generated synthetic data visually and statistically similar to the real data in an evaluation using the ICS dataset HAI and various indicators. The contributions of the paper are as follows:

- We extend the evidence lower bound of the variational inference to the time-series domain.
- We propose the attention-based variational recurrent autoencoder with the sequential evidence lower bound as the objective.
- We propose an algorithm for generating synthetic ICS time-series data using learned AVRAE.
- We comprehensively evaluate the quality of synthetic ICS time-series data generated by AVRAE in experiments using the HAI dataset.

The remainder of this paper is organized as follows. In Section 2, we present existing studies to generate synthetic data. Section 3 proposes the structure and learning algorithm of our proposal, AVRAE. Section 4 comprehensively evaluates AVRAE using an HAI [24], a widely used dataset collected from the HIL-based augmented ICS environment. In Section 5, some limitations of AVRAE are discussed. Finally, in Section 6, we describe future research along with conclusions.

## 2. Related Work

Many studies have been presented in machine learning and deep learning to generate data synthetically. This section presents data generation methods recently adopted in various fields, categorized into three categories. Section 2.1 introduces the variational-method-based data generation, closely related to our proposal. Section 2.2 and Section 2.3 present GAN and graph-based methods, respectively.

### 2.1. Variational Method-Based Generative Models

VAE [4] is a monumental model combining the variational method with the traditional autoencoder and allows for learning a powerful generative model (the decoder part). VAE learns the latent vectors’ probability distribution, representing the input data. Therefore, one can randomly generate the latent vectors that follow this probability distribution and generate realistic data through the decoder. VRAE [22] is a model combining the RNN with the variational method. This model successfully generated acoustic data through a dataset consisting of eight MIDI files. Yoo et al. [25] proposed a general variational-based generative architecture for augmenting datasets for understanding spoken language. Additionally, it was shown that the language understanding performance of the model was improved using the augmented dataset. The composed variational natural language generator (CLANG) [26] is a transformer-based conditional variational autoencoder that learns the latent representations of two properties (domain and action) of the intent. Through this, CLANG improved the performance of few-shot intent detection tasks. The variational transformer network (VTN) [27] was proposed to generate layouts, which are abstract locations between objects. This model has the ability to generate properties of the layout, such as margins and alignment, using the attention layer, which captures the relationships between objects within the layout as the building block.

### 2.2. GAN-Based Generative Models

GAN [5] is a framework for learning generative models proposed around the same time as VAE. GAN consists of the generator and the discriminator, and they learn competitively. VAE directly learns the distribution of the data (more precisely, the distribution of the latent variable), but due to its approximate approach, it cannot perfectly learn the data distribution. GAN [5] indirectly but sharply learns the data distribution. Chi et al. [28] presented a GAN model for generating time-series data in a smart grid. This model reparametrizes the energy consumption data to capture level information, and allows GAN to learn a conditional probability distribution to reflect pattern information. TimeGAN [7] is a variant of GAN specialized in generating time-series data and consists of the embedding function, the recovery function, the sequence generator, and the sequence discriminator. TimeGAN is flexible for multiple types of time-series data by simultaneously learning the static and dynamic characteristics of time-series data. CTGAN [8] successfully modeled the discrete and continuous columns in the tabular data and generated synthetic data. CTGAN performed better than the baseline (Bayesian network) on several benchmark datasets. TSGAN [29], like TimeGAN, was also proposed to generate time-series data. However, unlike TimeGAN, TSGAN focuses on one-dimensional data and adopts a few-shot approach.

### 2.3. Graph-Based Generative Models

A graph neural network (GNN) is a type of neural network designed to learn complex graph data structures. Recently, a graph convolutional network (GCN) has been adopted in many applications considering computational efficiency rather than naive GNN. D. Marcheggiani et al. [30] proposed a model generating text by processing graph data. This model consists of the graph convolutional encoder to process the graph structure of the input data and the LSTM-based decoder to generate text. The neural relational inference (NRI) [31] implicitly learns the graph structure underlying the data and predicts new data. While a typical GCN requires the adjacent matrix to process the graph structure of the data, NRI infers the graph structure solely from the observations. The spatio-temporal graph convolutional networks (STGCN) [32] considere both spatial and temporal dependency of data to predict mid- to long-term traffic volume. As a result, a competitive performance was shown compared with the baselines on various real-world traffic datasets.

## 3. Attention-Based Variational Recurrent Autoencoder

**Figure 1:**
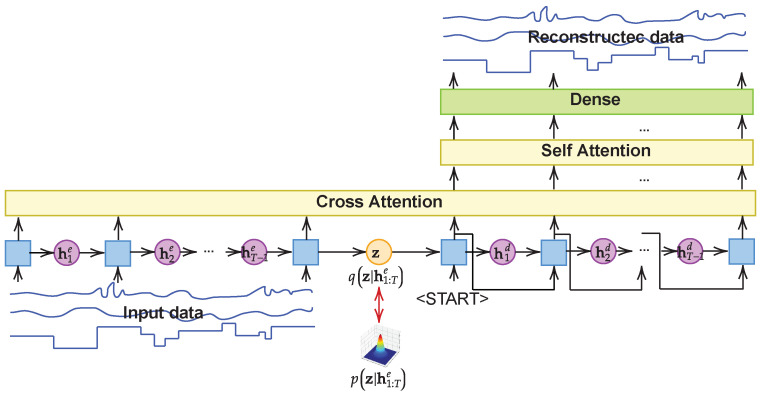
An architecture of the attention-based variational recurrent autoencoder.

In this section, we propose the attention-based variational recurrent autoencoder for generating time-series ICS data. Figure 1 shows the overall architecture of ARVAE. AVRAE consists of an RNN-based encoder and a decoder to process time-series data. Of course, RNN can receive input data at timestep *t* and immediately produce corresponding output data. However, through many studies in the past, it has been pointed out that such an architecture is disadvantageous when designing a prediction model for the sequential data [23]. Therefore, we adopted the encoder–decoder architecture, which creates a context that reflects the entire input sequence and can generate an output of variable length. As proposed in VRAE [22], the variational method is employed to learn the probability distribution over the context (the latent vector) in which the input sequence is summarized. Although this structure allows for learning a powerful generative model for time-series data, our preliminary experiments revealed that it cannot fully learn the characteristics of ICS time-series data. Variables in ICS time-series data include both discrete and continuous types. In addition, some variables have periodic short-term patterns, while others involve long-term dependencies. The preliminary experiments confirmed that VRAE is difficult to learn on such diverse data jointly. Therefore, we introduce the attention mechanism in VRAE to reflect both short- and long-term temporal patterns.

We formulate the sequential evidence lower bound to justify AVRAE in Section 3.1; In Section 3.2, the inference process in AVRAE is defined; Section 3.3 formulates the attention mechanism of AVRAE; Section 3.4 presents a learning algorithm for AVRAE; Finally, Section 3.5 presents the algorithm for generating synthetic ICS data.

### 3.1. Evidence Lower Bound

ARVAE is based on the variational inference seen in VAE [4], but unlike the original VAE, it deals with sequential data. Therefore, we first extend the variational inference’s basic evidence lower bound (ELBO) to the sequential data. The variational inference begins with approximating the true probability distribution p(z|x) of the latent variables z to the empirical probability distribution q(z), given the observations x.

(1) $$\begin{array}{cc} KL(q(z)||p(z|x))= & E_{q(z)}\left[\log q\left(z\right)\right]-E_{q(z)}\left[\log p\left(z,x\right)\right]+\log p\left(x\right) \end{array}$$

Equation (1) is the Kullback–Leibler divergence (KLD) between q(z) and p(z|x). The variational inference aims to find q(z), which minimizes Equation (1). However, in many cases, it is impossible to calculate p(z|x), so instead of directly minimizing it, KLD minimization is reduced to the problem of maximizing ELBO as follows, through further organizing.

(2) $$\begin{array}{cc} \log p(x)\geq & E_{q(z)}\left[\log p\left(z,x\right)\right]-E_{q(z)}\left[\log q\left(z\right)\right]\\ = & ELBO(q) \end{array}$$

Deriving Equation (2) from Equation (1) is fairly simple. We move KL(q(z)||p(z|x)) of Equation (1) to the right-hand side and logp(x) to the left-hand side. As KLD has a value greater than 0, logp(x) is always equal to ELBO(q). Basic ELBO does not address the sequentiality of the observations. Additionally, considering the latent variables between the observations in the time series, we extend ELBO as follows.

(3) $$\begin{array}{cc} ELBO(q)= & E_{q(z,h_{1:T}|x_{1:T})}\left[\log p\left(z,h_{1:T},x_{1:T}\right)\right]-E_{q(z,h_{1:T}|x_{1:T})}\left[\log q\left(z,h_{1:T}|x_{1:T}\right)\right]\\ = & E_{q(z,h_{1:T}|x_{1:T})}\left[\log p\left(x_{1:T}|z\right)\right]+E_{q(z,h_{1:T}|x_{1:T})}\left[\log p\left(z|h_{1:T}\right)\right]\\ & +E_{q(z,h_{1:T}|x_{1:T})}\left[\log p\left(h_{1:T}\right)\right]-E_{q(z,h_{1:T}|x_{1:T})}\left[\log q\left(z,h_{1:T}|x_{1:T}\right)\right]\\ = & E_{q(z,h_{1:T}|x_{1:T})}\left[\log p\left(x_{1:T}|z\right)\right]+E_{q(z,h_{1:T}|x_{1:T})}\left[\log p\left(z|h_{1:T}\right)\right]\\ & +E_{q(z,h_{1:T}|x_{1:T})}\left[\log p\left(h_{1:T}\right)\right]-E_{q(z,h_{1:T}|x_{1:T})}\left[\log q\left(z|h_{1:T}\right)\right]\\ & -E_{q(z,h_{1:T}|x_{1:T})}\left[\log q\left(h_{1:T}|x_{1:T}\right)\right]\\ = & E_{q(z,h_{1:T}|x_{1:T})}\left[\log p\left(x_{1:T}|z\right)\right]\\ & -KL(q\left(z|h_{1:T}\right)||p\left(z|h_{1:T}\right))-KL(q\left(h_{1:T}|x_{1:T}\right)||p\left(h_{1:T}\right)) \end{array}$$

Equation (3) is an extension of Equation (2) for the sequential data. To do this, we replace i.i.d observations x with the sequential observations $x_{1:T}$. Also, instead of adopting only a single latent variable z, which implies the semantics of all observations, we introduce the latent variable $h_{1:T}$, representing the sequentiality of the hidden states. Simplifying Equation (3) with Bayes rule and chain rule, ELBO consists of the expectation for the generation process, KLD for z, and KLD for $h_{1:T}$ (Hereafter, the word ELBO refers to Equation (3)). Therefore, to maximize ELBO, the two KLDs should be minimized while maximizing the expectation of the generation process. From this, we can derive the following facts:

- The probability distribution of the latent variables (or the hidden states) $h_{t}$ of each timestep *t* and the latent variables z of all $x_{t}$ and $h_{t}$ should be matched.
- The reconstruction objective as a generated model should be maximized.

These two conditions exactly match the process of encoder–decoder architecture, such as Seq2Seq. More specifically, the above ELBO can be optimized by learning the probability distribution for $h_{t}$ and the context passed from the encoder to the decoder at each timestep of the RNN and training the entire model with the autoencoder. We use this ELBO as the objective to learn AVRAE.

### 3.2. Inference Process

The inference of AVRAE combines the encoder–decoder model and VAE. More specifically, the encoder and the decoder follow general RNN operations, but two modifications are applied. First, the hidden state $h_{t}$ of each timestep should follow a stochastic process. Because general RNN operations are deterministic, they cannot satisfy ELBO. Therefore, we sample the hidden state $h_{t}$ from a probability distribution instead of computing it deterministically. From this, the feedforward of the encoder and the decoder is defined as Equation (4).

(4) $$h_{t}∼q_{\theta }\left(h_{t}|h_{<t-1},x_{<t}\right)$$

where $h_{<t-1}$ and $x_{<t}$ refer to all the hidden states up to the timestep t−1 and all the observations up to the timestep *t*. $q_{\theta }$ is a probability distribution parameterized by θ. We adopt the Markov property in Equation (4) to assume that the hidden state $h_{t}$ at the timestep *t* depends only on the hidden state of the previous timestep and the observation of that timestep.

(5) $$h_{t}∼q_{\theta }\left(h_{t}|h_{t-1},x_{t}\right)$$

where $x_{t}\in R^{p}$ and $h_{t}\in R^{p}$ are the observation and the hidden state, respectively, at timestep *t*, although various probability distributions can be employed as $q_{\theta }$, in this paper we simply adopt a standard Gaussian distribution as $q_{\theta }$. Through this sampling, a non-differential operation can be easily replaced through the reparameterization trick [4,22,33].

(6) $$h_{t}∼N\left(\mu_{h_{t}},\mathrm{diag}\left(\sigma_{h_{t}}^{2}\right)\right),\;\mathrm{where}\;\left[\mu_{h_{t}},\sigma_{h_{t}}\right]=\phi \left(h_{t-1},x_{t}\right)$$

Similarly, the latent variable z (or the context) is passed from the encoder to the decoder and is sampled according to the variational inference. However, the context depends on the hidden state generated at the last timestep of the encoder.

(7) $$z∼q_{w}\left(h_{T}\right)$$

where $z\in R^{r}$ is the context in which all sequence information is summarized. $q_{w}$ is a probability distribution parameterized by *w*, and various probability distributions can be considered for this. But, like $q_{\theta }$, we use a standard Gaussian distribution as $q_{w}$.

(8) $$z∼N\left(\mu_{z},\mathrm{diag}\left(\sigma_{z}^{2}\right)\right),\;\mathrm{where}\;\left[\mu_{z},\sigma_{z}\right]=\psi \left(h_{T}\right)$$

Finally, the decoder uses the context z as the initial hidden state and the same feedforward operation as the encoder. The only difference from the encoder is that the decoder does not use the actual observations as the input data because it should be trained as a generative model but it always adopts the output of the previous timestep as the input data. Also, the decoder output should be the same as the encoder’s input.

### 3.3. Attention Layer

It is well known through various studies [1,20] that the attention mechanism brings benefits to the prediction performance of models handling time-series data. In particular, the attention mechanism exerts a significant effect on the encoder–decoder model. In the traditional Seq2Seq, the most representative model of the encoder-decoder structure, the decoder produces an output using only a fixed-size vector (the context) generated by the encoder. In this structure, the last output is extremely far from the first input, making it difficult to completely convey the input information to the decoder with only the context. Therefore, in this paper, we introduce the attention layer to allow the decoder part of AVRAE to focus on the necessary parts of the input sequence when generating the output sequence. AVRAE employs two types of attention layers: cross-attention and self-attention.

**Cross-attention**. The cross-attention models the correlation between the encoder’s output sequence and the decoder’s output sequence. This allows us to learn which parts of the input sequence the decoder’s output should depend on (strictly speaking, we utilize the hidden states of the encoder and the decoder to calculate the attention weights). Additionally, we used a look-ahead mask to ensure that the decoder is not influenced by the preceding sequence when generating the output.

(9) $$\begin{array}{cc} W_{c}= & \mathrm{softmax}(\frac{H_{dec}H_{enc}^{T}}{\sqrt{d}}+\left(1-M\right)\times -\infty )\\ O_{c}= & LN(MLP\left(W_{c}H_{enc}\right)+H_{enc}) \end{array}$$

where $H_{enc}\in R^{T\times d}$ and $H_{dec}\in R^{T\times d}$ are the hidden states of all timesteps of the encoder and the decoder, respectively. $M\in {\left\{0,1\right\}}^{T\times T}$ is a look-ahead mask. $O_{c}\in R^{T\times d}$ is the output of the cross-attention. The attention weights $W_{c}$ are calculated through the softmax function after applying a mask to the normalized attention score. The value multiplied by $W_{c}$ and $H_{enc}$ is transformed through multi-layer feedforward perceptron (MLP) and added to $H_{enc}$ through a skip connection. Then, the output $O_{c}$ of the cross-attention is produced by applying the layer normalization (LN) [34].

**Self-attention**. $O_{c}$ produced through the cross-attention layer is further emphasized by the self-attention layer. The self-attention layer has similar computations to the cross-attention layer.

(10) $$\begin{array}{cc} W_{s}= & \mathrm{softmax}(\frac{O_{c}O_{c}^{T}}{\sqrt{d}})\\ O_{s}= & LN(MLP\left(W_{s}O_{c}\right)+O_{c}) \end{array}$$

where $O_{s}\in R^{T\times d}$ is the output of the self-attention layer. As shown in Equation (10), the self-attention layer, unlike the cross-attention layer, does not use masking and only calculates using the same type of input $O_{c}$.

Finally, the output $O_{s}$ of the self-attention layer is converted to the same shape as the encoder’s input through the dense layer and becomes the decoder output.

### 3.4. Learning Process

In this Section, we present the learning process of AVRAE, combining all previously presented components. AVRAE has an encoder-decoder architecture similar to Seq2Seq. Therefore, the inference procedures of the encoder and the decoder are presented first, and then we combine them to present the learning algorithm.

Algorithm 1 is the feedforward process of the encoder. This algorithm takes a minibatch $x_{1:T}$ of size *m* as the input and returns $H_{e}nc$ and Λ, z, $\left[\mu_{z},\sigma_{z}\right]$. Where Λ is the set of all parameters of the Gaussian distribution of hidden states. This algorithm is extremely simple. The recurrent operation $\phi_{e}$ is performed with the input data $x_{t}$ of every timestep *t* and $h_{t-1}^{e}$, the hidden state of the previous timestep. $\phi_{e}$ produces $\left[\mu_{h_{t}}^{e},\sigma_{h_{t}}^{e}\right]$, and these are employed as parameters of the Gaussian distribution to sample the hidden state $h_{t}^{e}$ of the next timestep. After performing a recurrent operation on the input $x_{t}$ of all timesteps, the parameters $\left[\mu_{z},\sigma_{z}\right]$ of the Gaussian distribution for sampling the latent variable z from the hidden state $h_{T}^{e}$ of the last timestep *T* are produced.
**Algorithm 1:** A procedure for the encoder
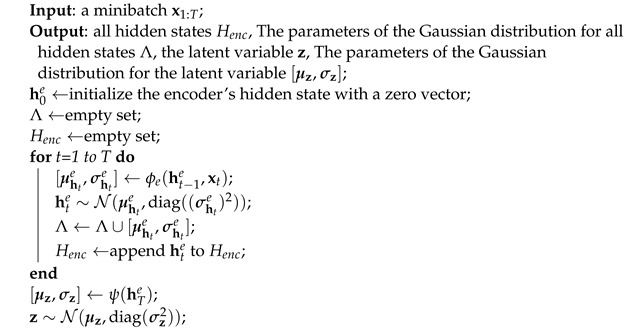


Algorithm 2 is the feedforward procedure of the decoder. To perform the decoding operation of AVRAE, this algorithm receives all the hidden states $H_{enc}$ and the latent variable z generated by the encoder as the input. It returns the reconstructed data ${\hat{x}}_{1:T}$. The initial hidden state of the decoder is initialized with the latent variable z. As described in Section 3.3, the decoder should be trained as a generative model. Therefore, rather than using the actual observations as the input, the decoder takes the output $x_{t-1}^{d}$ of the previous timestep generated by the recurrent operations as the input. For this purpose, the decoder simply adopts a symbol indicating the <START> of the sequence as the input of the first timestep, and, in this paper, a zero vector is used. Like the encoder, the decoder repeats the same calculation using the recurrent operation $\phi_{d}$ and collects the hidden state at each timestep *t* to create $H_{dec}$. Then, $O_{s}$ is produced by sequentially calculating the cross-attention and self-attention using the encoder’s hidden states $H_{enc}$ and the decoder’s $H_{dec}$. Finally, the reconstructed data ${\hat{x}}_{1:T}$ are generated from $O_{s}$ through the dense layer.

Algorithm 3 trains AVRAE by combining the encoder and decoder procedures presented previously. First, the algorithm samples a minibatch $x_{1:T}$ of size *m* from a training dataset D. The encoder procedure processes this $x_{1:T}$ to produce $H_{enc}$ and $\Lambda ,z,[\mu_{z},\sigma_{z}]$. The decoder reconstructs ${\hat{x}}_{1:T}$ using $H_{enc}$ and z. Finally, this algorithm calculates the loss according to the ELBO formulated in Equation (3), calculates the gradients from this, and updates the parameters of AVRAE. To compute the loss function on a minibatch basis, we slightly abuse the notation for ELBO in this algorithm. Where $ELBO(q_{i})$ is the ELBO calculated for the *i*-th observation included in the minibatch.
**Algorithm 2:** A procedure for the decoder
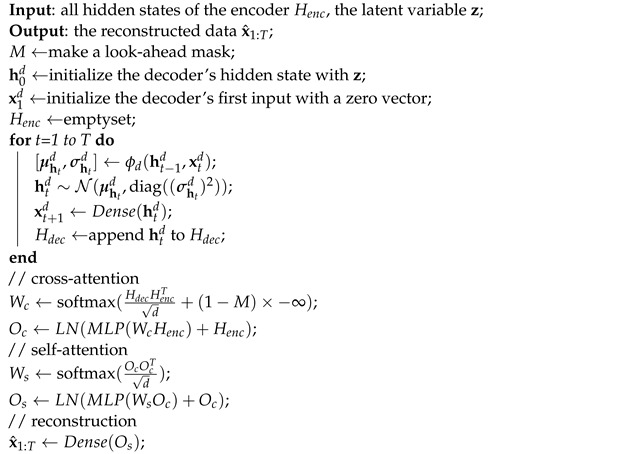


**Algorithm 3:** A learning algorithm for AVRAE.


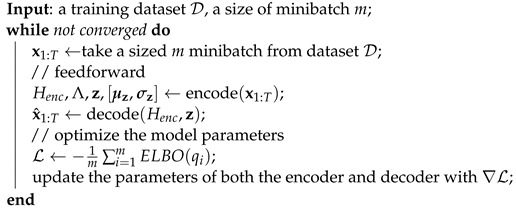




### 3.5. Generation Process

As a general VAE learns only the distribution of the latent variables, which are the output of the encoder, only the decoder can be used to generate data if only this distribution is known. However, because AVRAE proposed in this paper deals with sequential data and uses two latent variables, it is difficult to generate synthetic data simply using the decoder (in particular, even if the hidden state $h_{t}$ is trained to follow a standard Gaussian distribution, sampling is difficult because it is influenced by the observations and hidden states of past timesteps). Therefore, instead of generating data using only the learned decoder, we utilize both the encoder and the decoder. Additionally, instead of generating entirely new data, we adopt a strategy that utilizes the seed input.

Algorithm 4 is an algorithm for generating synthetic time-series ICS data using AVRAE. This algorithm is straightforward but effective in generating synthetic data. The algorithm’s inputs are the learned ARVAE (the encoder and the decoder), the number of data to be generated *N*, and a real dataset D. A real dataset D does not matter whether it is a training dataset or not. As demonstrated in VAE [4], the variational lower bound allows for learning an unbiased estimator. First, one sequential observation is taken from dataset D as a seed. The encoder produces the mean $\mu_{h_{t}}$ and standard deviation $\sigma_{h_{t}}$ for the hidden states at all timesteps for the observation and the mean $\mu_{z}$ and standard deviation $\sigma_{z}$ for the context. Then, $H_{enc}^{i}$ and $z^{i}$ are sampled from the Gaussian distribution. The decoder uses the sampled $H_{enc}^{i}$ and $z^{i}$ to generate synthetic ICS data ${\hat{x}}_{1:T}^{i}$. The generated data are collected in Δ. We can repeat the above process *N* times to generate as much synthetic data as desired. This algorithm is a stochastic process that introduces uncertainty while sampling $H_{enc}$ and $z^{i}$. Therefore, the generated synthetic data all have different values but have a modality similar to the seed due to the structural characteristics of the encoder–decoder.
**Algorithm 4:** Synthetic ICS time-series data generation using AVRAE.
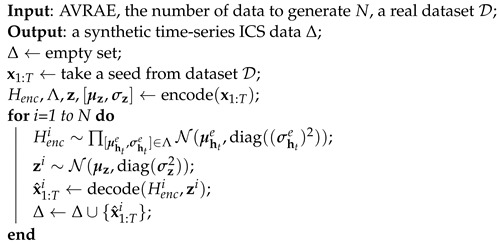


## 4. Evaluation

In this section, we evaluate AVRAE proposed in Section 3 using a benchmark dataset. Section 4.1 describes the ICS dataset used in the experiment; Section 4.2 covers the experimental environment, model structure, and preprocessing; Section 4.3 evaluates the quality of data generated by AVRAE through various performance indicators; Section 4.4 assesses the roles of the AVRAE’s components; Section 4.5 compares AVRAE with several baselines.

### 4.1. Dataset Description

Because ICS systems are generally operated in a closed environment due to security and privacy issues, datasets collected from actual ICS environments are scarce. However, in several studies, the data collected by building an ICS testbed were made public, and we conducted experiments with the HAI dataset [24]. This dataset was collected from a water treatment testbed. The testbed consists of a turbine, boiler, and water treatment system integrated into the hardware-in-the-loop (HIL) simulator. As the HAI testbed is regularly updated, datasets are periodically published. HAI 23.05 has been released, and this dataset consists of a training dataset collected for 249 h and a test dataset collected for 79 h. The training dataset contains only normal data, and the test data contain 52 attacks.

### 4.2. Implementation Details

**Computational environment**. We performed all experiments in the same computational environment: Intel(R) Core(TM) i9-11900 2.50 GHz, 32 GB RAM, and Ubuntu 20.04 LTS 64-bit. Additionally, NVIDIA GTX 3080 Titan was used for efficient model learning as VGA hardware acceleration.

**Hyper-parameter settings**. AVRAE in the experiment was implemented as a stacked LSTM-based RNN [18]. AVRAE’s encoder and decoder are each built with two layers, and *p*, the size of the observation $x_{t}$ at timestep *t*, is 48. The number of features included in the original HAI dataset is over 80. Among these, we used only 48 features as the input data, excluding some features with constant values and features with missing values during preprocessing). Additionally, *d*, the size of the hidden state $h_{t}$, was set to 1024, and *r*, the size of the context z, was set to 64. The layer normalization [34] was applied between each layer of the stacked RNN. AVRAE was learned with the Adam optimizer [35], and $5\times 10^{-4}$ was used as the learning rate.

**Preprocessing**. Each feature in the HAI dataset has a different value range. Because this type of data are detrimental to model learning, we made the scale of the data consistent. Generally, min–max scaling and standardization are used. Because the features of the HAI dataset often have unclear or meaningless upper and lower bounds for features, we adopted standardization. Additionally, the HAI dataset contain data collected over a long period of time. However, because data should be expressed in shorter units for input to AVRAE, we used 100 data at a time as input to AVRAE (i.e., T=100).

### 4.3. Quality Assessment

In this section, we measure the quality of ICS time-series data produced by AVRAE through various figures and indicators. More specifically, this experiment employed plotting of original and generated data, manifold, dynamic time warping (DTW) [36], mean absolute percentage error (MAPE), and kernel density estimation (KDE).

**Figure 2:**
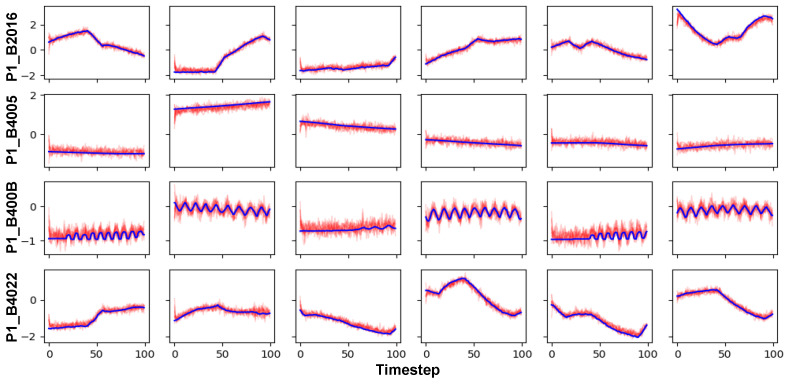
A visualization for real and synthetic data.

Figure 2 visualizes real HAI data and synthetic data generated by AVRAE. In this figure, only six features showing long-term and short-term temporal patterns are expressed instead of all features (P1_B2016, P1_B4005, P1_B400B, P1_B4022). In the figure, the blue lines represent the real data and the red lines represent synthetic data. In addition, 10 synthetic data were generated for each single seed according to Algorithm 4. Overall, synthetic data are clustered in a similar pattern near the real data used as seed. Additionally, synthetic data exhibit uncertainty because AVRAE follows the stochastic process described in Section 3. One thing to note is that the position of the point of the first timestep of the synthetic data is somewhat different from that of the seed. This is because we used the zero vector as the ‘<START>’ symbol as the input of the first timestep of the decoder. Excluding these minor errors, it was confirmed that AVRAE successfully jointly learned long-term and short-term time-series patterns overall.

**Figure 3:**
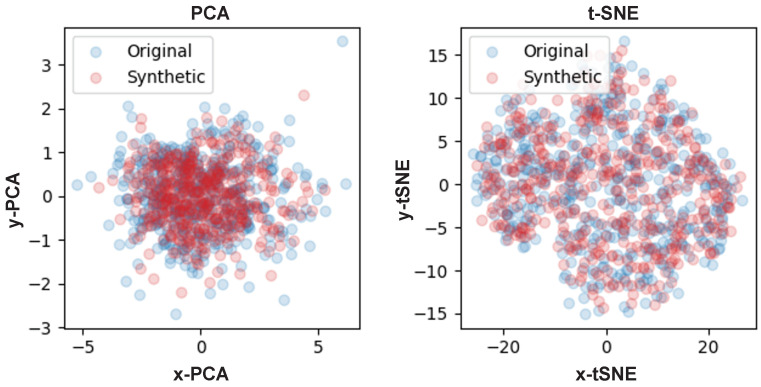
A visualization for PCA (**left**) and t-SNE (**right**).

The plausibility of the synthetic data was confirmed through visualization in Figure 2. At this time, we checked whether this data show a similar distribution to the real data in a manifold space. Figure 3 is a visualization using principal component analysis (PCA) and t-distributed stochastic neighbor embedding (t-SNE) [37] for the real data and synthetic data. PCA reduced the dimensionality while preserving the variance of high-dimensional data. On the other hand, t-SNE preserved the topology of high-dimensional data while emphasizing spatial separation between data clusters. In other words, data in high-dimensional space were located close to the manifold. In the figure, blue dots represent the real data and red dots represent the synthetic data. Of course, PCA is not a suitable algorithm for visualizing high-dimensional data, but it allows for identifying patterns and variability (i.e., variance) in the data. Through experiments with PCA, we confirmed that the real and synthetic data have similar variances. The same result was confirmed in experiments with t-SNE. Considering that the real data and the synthetic data were embedded in the same space, it can be said that it was difficult to distinguish between the two data.

**Figure 4:**
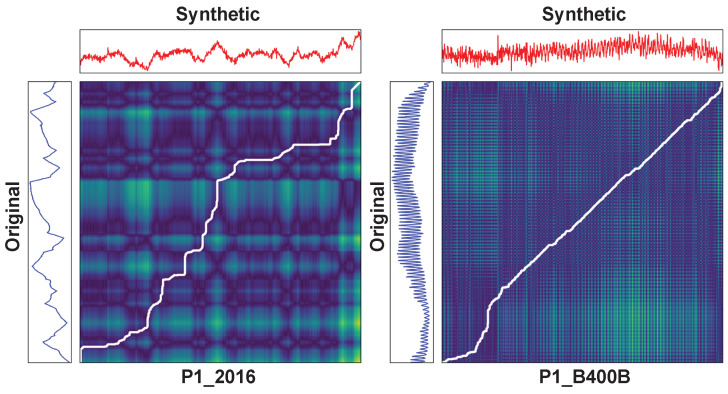
A visualization for dynamic time warping between the real and synthetic data. (**left**): P1_2016, (**right**): P1_B400B.

Figure 4 shows the similarity between the real and synthetic data using DTW. DTW is an algorithm that measures the similarity between two time-series data. This algorithm searches for the optimal matching path between two-time-series data. We selected two features and compared them with DTW to show that AVRAE learned both short- and long-term temporal patterns well. P1_2016 was chosen as a feature showing a long-term pattern, and P1_B400B was selected as a feature showing a short-term pattern. In Figure 4, the blue line on the left is the real data, the red line on the top is the synthetic data, and the matrix in the center is the similarity matrix. The white line in the similarity matrix is the so-called warping path. The warping path is a set of points that represents the optimal match between two time-series data. In other words, the closer this white line is to the diagonal line running from the bottom left to the top right of the similarity matrix, the more similar the data being compared are. As shown in Figure 4, it can be seen that both the data with short- and long-term patterns generated by AVRAE are quite similar to the real data.

**Figure 5:**
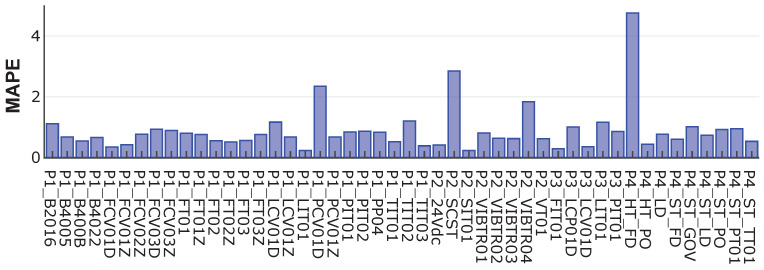
Mean absolute percentage error for every feature between the real and synthetic data.

Figure 5 shows the MAPE between the real data and the data generated by AVRAE. MAPE represents the average absolute percent error between the predicted and actual values. MAPE is not affected by the scale of the data, and because it expresses the error as a percentage, it is easy and intuitive to interpret. As can be seen from the experimental results, the MAPE for most features is less than 1%, and the feature with the largest difference does not exceed 5%. In other words, AVRAE generates data with sufficient uncertainty while the generated data do not deviate significantly from the seed.

**Figure 6:**
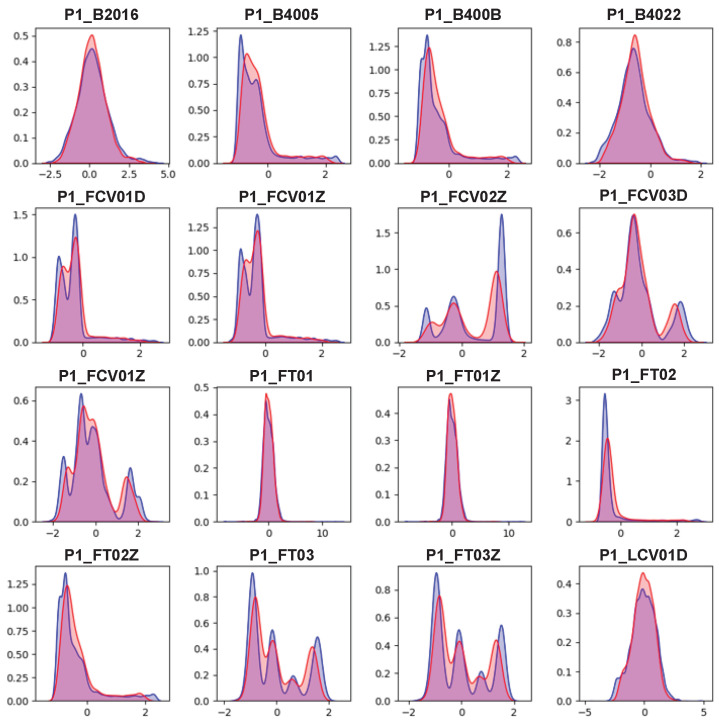
Kernel density estimation between the real and synthetic data.

Finally, we checked the distribution of values for each feature in the real and synthetic data. Figure 6 visualizes the distribution of values of the real and synthetic data using KDE. In the figure, the blue area is the distribution of the real data and the red area is the area of the synthetic data. Because we adopted standardization as preprocessing, most feature values were centered around 0 (average). As shown in the figure, most features of the real data have multi-modal characteristics. Traditionally, the Gaussian mixture model (GMM) is used to model such data. However, GMM requires pre-specifying the number of Gaussian distributions to be modeled, and the computational complexity increases as the number of data increase. In comparison, AVRAE excellently approximated the multi-modal distribution of the real data without explicitly specifying the number of distributions to be modeled.

### 4.4. Ablation Study

We performed an ablation study to analyze the role of each component of AVRAE, such as the hidden state sequence $h_{1:T}$, the latent variable z, and the attention mechanism.

**Figure 7:**
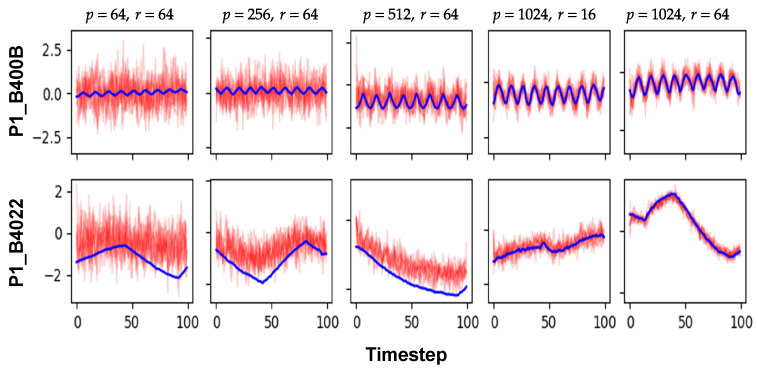
Visual comparison according to the sizes of the hidden states and the latent variable.

**Figure 8:**
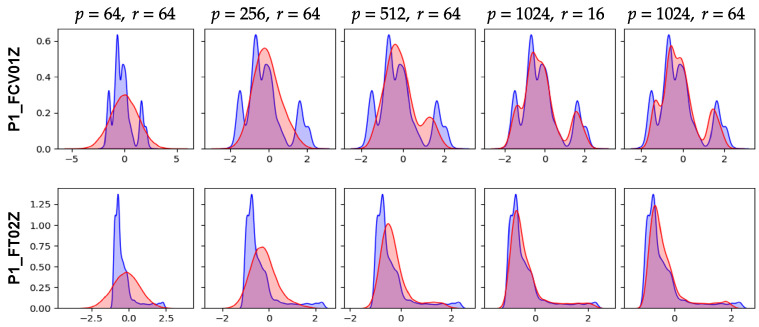
KDE comparison according to the sizes of the hidden states and the latent variables.

Figure 7 and Figure 8 are comparisons of the synthetic ICS data according to the sizes of the hidden states and the latent variables (*p* and *r*, respectively). The first three columns in Figure 7 show the modality of the synthetic data according to *p* when *r* is 64. Clearly, for smaller *p*, AVRAE generated the pure stationary data. In other words, if the capacity of the hidden state was not sufficient, AVRAE could not fully learn the characteristics of the time-series data. Next, the two right columns of Figure 7 show the difference in synthetic data according to *r* when *p* is 1024. In both settings, AVRAE learned the short-/long-term patterns of the ICS data well. However, when *r* was small, it was observed that the variance of the generated data was somewhat large.

The above observation was confirmed once again through Figure 8. In Figure 7, the smaller *p*, the data were close to the stationary distribution. In Figure 7, it was confirmed that the values of the features actually formed a Gaussian distribution. This is the effect of ELBO, which used the same Gaussian distribution as the true distribution, although the dynamics of the ICS data could not be learned because the capacity of the hidden state was not sufficient. In fact, as *p* increased, AVRAE learned the distribution of the actual data more precisely. On the other hand, when *p* was large enough, the difference by *r* was not noticeable.

**Figure 9:**
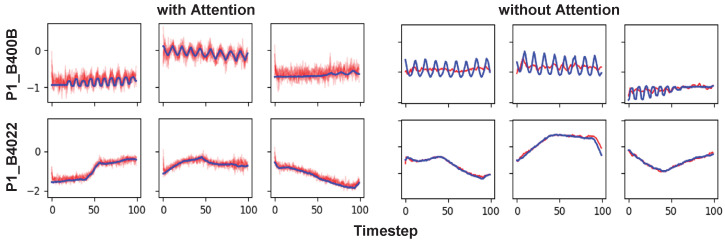
Visual comparison of the synthetic ICS data according to the attention mechanism.

Figure 9 is a visual comparison of AVRAE’s time-series data generation according to the attention mechanism. In the figure, the three left columns are data generated by AVRAE with the attention mechanism enabled. The three columns on the right are data generated by AVRAE with the attention mechanism disabled (technically, a model that disables the attention mechanism cannot be called AVRAE. However, for convenience of explanation, we refer to this setting as ‘AVRAE without the attention’). The experimental results showed that the attention layers had a significant impact on data generation. First, the most notable feature of AVRAE without the attention was that the generated data only learned the long-term trends. P1_B400B of the HAI dataset contained time-series patterns with short periods. However, when the attention was disabled, the model had difficulty learning such short-term dependency. This means that, according to our design, the attention layers effectively reflected the characteristics of the encoder’s input time series $x_{1:T}$ to the decoder’s output. Another interesting fact about the attention layers is the uncertainty of the generated data. Intuitively, the data generated by AVRAE with the attention have a higher variance. This is related not only to the attention, but also to the data generation algorithm presented in Section 3.5. Algorithm 4 generates new $H_{enc}^{i}$ and $z^{i}$ each time using Λ and $\left[\mu_{z},\sigma_{z}\right]$ from the encoder processing the seed. In other words, the uncertainty derived from the sampling process for the hidden states and the latent variable was better reflected in the decoder’s output by the attention layers. The observations imply that AVRAE is a suitable model for generating ICS time-series data.

### 4.5. Comparative Study

In this section, we compare AVRAE with the baseline models. TimeGAN [7] and VRAE [22] were selected as baselines for the comparative study. The reasons these two models were selected as baselines were as follows: TimeGAN is a representative time-series generation model designed based on GAN; VRAE is the variational inference-based sequential autoencoder and is the direct parent of AVRAE. We built TimeGAN and VRAE with a similar complexity to AVRAE in Section 4.3 for comparative experiments. TimeGAN’s hidden state size was set to 1024 and optimized by the Adam optimizer (with learning rate $5\times 10^{-4}$. In VRAE, the size of the hidden state was set to 1024 and the size of the latent variable between the encoder and the decoder was set to 64. VRAE was also trained by the Adam optimizer (with a learning rate $5\times 10^{-4}$. For comparison between models, visualization between the synthetic data and real data, manifold space, and KDE were used.

**Figure 10:**
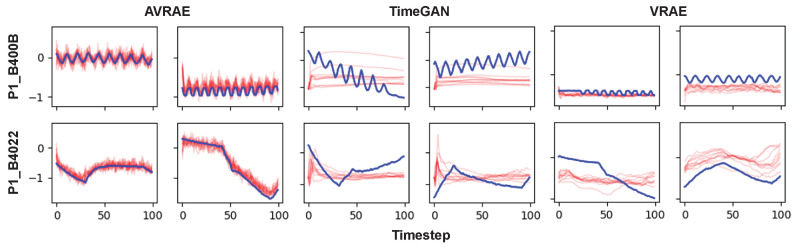
Visual comparison between generative models. The first two columns belong to AVRAE. The middle two columns’ data are generated by TimeGAN. The last two columns’ data are produced by VRAE.

Figure 10 shows the synthetic ICS data and the real data generated by AVRAE and baselines. The meaning of the line colors is the same as in Figure 2. AVRAE and VRAE generated the synthetic data basically following Algorithm 4. Because VRAE has a similar structure to AVRAE, it can generate data almost the same way, with the only differences being in the attention mechanism and the sampling process for the hidden states. As shown in the figure, TimeGAN failed to generate intuitively meaningful time-series data. More specifically, TimeGAN did not sufficiently capture the long short-term dependencies or dynamics inherent in ICS data. VRAE learned the dynamics of ICS data well compared with TimeGAN, but it was not accurate. The data generated by VRAE are also quite distant from the seed input. AVRAE simultaneously learned the time dependency and dynamics of ICS data compared with the baselines.

**Figure 11:**
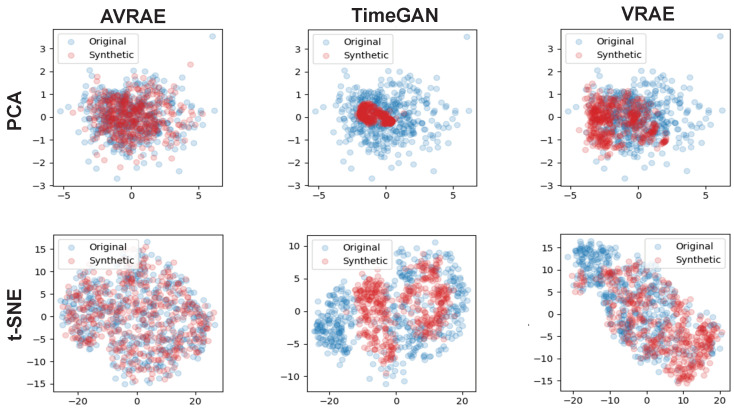
Comparison in the manifold space between the generative models.

Figure 11 compares the synthetic time-series and the real data generated by AVRAE, TimeGAN, and VRAE in the manifold space. In experiments using PCA, it was confirmed that TimeGAN did not sufficiently represent the variance of the real data. VRAE was better than TimeGAN, but it could not represent some data. This is also clearly evident in comparison through t-SNE. TimeGAN failed to represent the real data overall. VRAE had a similar distribution to the real data, but was analyzed to be somewhat overfitted. Compared with other models, the synthetic data generated by AVRAE were almost identical to the real data in terms of data distribution in manifold space.

**Figure 12:**
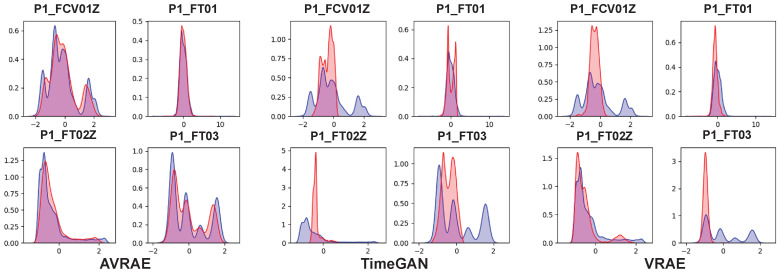
Comparison in KDE between the generative models.

Figure 12 compares the synthetic and real data generated by AVRAE, TimeGAN, and VRAE at the feature level. The color code is the same as in Figure 6. Although the distribution of data generated by TimeGAN was very sharp, it represented only a portion of the real data. This is a typical phenomenon observed in GAN-based generative models. GAN tends to implicitly but very sharply learn the real data distribution. On the other hand, VRAE learned the distribution of the real data better than TimeGAN overall, but showed a tendency to be overfitted. More specifically, the data generated by VRAE were concentrated near the mode of the real data distribution. The data generated by AVRAE followed the distribution of the real data well without bias.

There are two main reasons AVRAE can generate better-quality data than other baselines. First, compared with VRAE, which uses a single latent variable for the entire observations, AVRAE adopts sequential latent variables for the hidden states. This allows the dynamics contained in ICS data to be expressed more flexibly. Second, using the attention mechanism, AVRAE captures ICS data’s time dependency better and learns the data distribution without bias.

## 5. Limitations

Although AVRAE proposed in this paper successfully generates synthetic ICS time-series data, it still has some limitations. This section discusses these limitations and presents potential insights to address them.

- The AVRAE-based data generation algorithm proposed in this paper uses real data as a seed. The data generated as a result show a pattern similar to the seed. As a result, the generated data are seed-dependent and lack diversity. Of course, the diversity of data can be secured by controlling the variance when sampling the hidden states and the context in the inference process. However, the data generated this way are closer to anomaly than normal. One alternative is to learn a sequential probability distribution of the hidden states. The hidden markov model (HMM) is one of the most suitable models for this task. However, as the dimensionality of HMM increases, the computational complexity increases significantly. Conversely, if the dimensionality of the hidden states in AVRAE is set small to keep the computational complexity low, the hidden states may not fully reflect the dynamics of the observations. The seed issue is a problem that cannot be easily solved, and approaches from various aspects other than HMM are required.
- Another cause of the lack of diversity in the generated data is that AVRAE is learned using an autoencoder method. We can overcome this limitation by training AVRAE as a forecasting model. In other words, AVRAE’s decoder learns to predict data a few steps later than the observations that are input to the encoder. Through this, the constraint of the autoencoder that input–output must be the same is resolved, allowing AVRAE to generate more diverse data.
- The generated data contain noise resulting from the sampling of the latent variables. Because of this, the synthetic data are somewhat messy compared with the real data. This can be overcome through smoothing methods such as moving averages. This can be overcome mainly in two ways. First, use a smoothing technique such as the moving average. Simply, a moving average slides a window and calculates the average of several observations. The second method is to adopt the expected value by generating multiple independent time-series samples. The above techniques basically remove the uncertainty of a single observation.

## 6. Conclusions

In this paper, we extend the evidence lower bound of the variational inference to time-series data. We also proposed the variational recurrent autoencoder, which learns this as an objective. This model uses an attention mechanism to jointly learn ICS time-series data’s short- and long-term temporal dependencies. Additionally, a synthetic data generation algorithm using learned AVRAE was proposed. In comprehensive experiments using HAI, a well-known ICS dataset, we confirmed that ARVAE can successfully generate synthetic ICS time-series data. The quality of the generated data was checked visually through manifold, DTW, MAPE, and KDE. Finally, some limitations of AVRAE were discussed.

This study was conducted to resolve data scarcity and can be utilized in the ICS environment in various aspects. First, AVRAE can contribute to checking the stability of the control system by generating synthetic time-series data. Control systems contain complex control logic. Verifying the soundness of this control logic is an essential safety-related issue, but there are limits to verifying the logic with limited data. Therefore, the reliability of the control system can be improved by generating various data through AVRAE and using it for verification. Second, AVRAE can be used to generate abnormal data. Anomaly detection is an open problem in the security domain. Recently, research on machine-learning-based anomaly detection systems has been actively conducted. However, due to the nature of the ICS environment, where it is difficult to obtain abnormal data, it is difficult to learn a robust detection model. By introducing AVRAE into anomaly data generation, a more reliable detection model can be learned, which is also our future work.

AVRAE has successfully generated synthetic data, but the diversity of the generated data are somewhat low due to limitations arising from autoencoder learning. However, this research has laid the foundation for learning more powerful generative models for time-series data. In the future, we will conduct research to overcome the limitations discussed here. More specifically, we will study a complete generative model and its process so that the synthetic data generated do not depend on the seed. Secondary research, such as abnormal data generation, will also be performed.

## Data Availability

Data are contained within the article.

## Abbreviations

The following abbreviations are used in this manuscript:

ICS	Industrial Control System
AVRAE	Attention-based variational recurrent autoencoder
VAE	Variational autoencoder
GAN	Generative adversarial networks
i.i.d	Independently-and-identically distributed
RNN	Recurrent neural network
LSTM	Long short-term memory
GRU	Gated recurrent unit
VRAE	Variational recurrent autoencoder
Seq2Seq	Sequence to Sequence
CLANG	Composed variational natural language generator
VTN	Variational transformer network
GNN	Graph neural network
GCN	Graph convolutional network
STGCN	Spatio-temporal graph convolutional network
ELBO	Evidence lower bound
KLD	Kullback-Leibler divergence
MLP	Multi-layer perceptron
LN	Layer normalization
HIL	Hardware-in-the-loop
DTW	Dynamic time warping
MAPE	Mean absolute percentage error
KDE	Kernel density estimation
PCA	Pincipal component analysis
t-SNE	t-distributed stochastic neighbor embedding
GMM	Gaussian mixture model
HMM	Hidden markov model

## References

[1] Vaswani A., Shazeer N., Parmar N., Uszkoreit J., Jones L., Gomez A.N., Kaiser Ł., Polosukhin I. (2017). Attention is all you need. arXiv.

[2] Radford A., Narasimhan K., Salimans T., Sutskever I. (2018). Improving Language Understanding by Generative Pre-Training. OpenAI.Com. https://openai.com/research/language-unsupervised.

[3] Radford A., Narasimhan K., Salimans T., Sutskever I. (2019). Language Models Are Unsupervised Multitask Learners. OpenAI Blog. https://cdn.openai.com/better-language-models/language_models_are_unsupervised_multitask_learners.pdf.

[4] Kingma D.P., Welling M. Auto-encoding variational bayes. Proceedings of the 2nd International Conference on Learning Representations, ICLR 2014—Conference Track Proceedings.

[5] Goodfellow I.J., Pouget-Abadie J., Mirza M., Xu B., Warde-Farley D., Ozair S., Courville A., Bengio Y. Generative adversarial nets. Proceedings of the Advances in Neural Information Processing Systems.

[6] Wan Z., Zhang T., He H. Variational Autoencoder Based Synthetic Data Generation for Imbalanced Learning. Proceedings of the 2017 IEEE Symposium Series on Computational Intelligence (SSCI).

[7] Yoon J., Jarrett D., van der Schaar M. Time-series generative adversarial networks. Proceedings of the 33rd Conference on Neural Information Processing Systems (NeurIPS 2019).

[8] Xu L., Skoularidou M., Cuesta-Infante A., Veeramachaneni K. Modeling tabular data using conditional GAN. Proceedings of the Advances in Neural Information Processing Systems.

[9] Zhu J.Y., Park T., Isola P., Efros A.A. Unpaired image-to-image translation using cycle-consistent adversarial networks. Proceedings of the IEEE International Conference on Computer Vision.

[10] Rebuffi S.A., Gowal S., Calian D., Stimberg F., Wiles O., Mann T. (2021). Data Augmentation Can Improve Robustness. arXiv.

[11] Zhong Z., Zheng L., Kang G., Li S., Yang Y. Random Erasing Data Augmentation. Proceedings of the AAAI Conference on Artificial Intelligence.

[12] Devlin J., Chang M.W., Lee K., Toutanova K. BERT: Pre-training of deep bidirectional transformers for language understanding. Proceedings of the NAACL HLT 2019—2019 Conference of the North American Chapter of the Association for Computational Linguistics: Human Language Technologies-Proceedings of the Conference.

[13] Ouyang L., Wu J., Jiang X., Almeida D., Wainwright C.L., Mishkin P., Zhang C., Agarwal S., Slama K., Ray A. Training language models to follow instructions with human feedback. Proceedings of the Advances in Neural Information Processing Systems.

[14] Hannun A., Case C., Casper J., Catanzaro B., Diamos G., Elsen E., Prenger R., Satheesh S., Sengupta S., Coates A. (2014). Deep Speech: Scaling up end-to-end speech recognition. arXiv.

[15] Chorowski J., Bahdanau D., Serdyuk D., Cho K., Bengio Y. Attention-based models for speech recognition. Proceedings of the Advances in Neural Information Processing Systems.

[16] Donahue J., Hendricks L.A., Rohrbach M., Venugopalan S., Guadarrama S., Saenko K., Darrell T. (2017). Long-Term Recurrent Convolutional Networks for Visual Recognition and Description. IEEE Trans. Pattern Anal. Mach. Intell..

[17] Ng J.Y.-H., Hausknecht M., Vijayanarasimhan S., Vinyals O., Monga R., Toderici G. Beyond short snippets: Deep networks for video classification. Proceedings of the IEEE Computer Society Conference on Computer Vision and Pattern Recognition.

[18] Hochreiter S., Schmidhuber J. (1997). Long short-term memory. Neural Comput..

[19] Cho K., van Merriënboer B., Gulcehre C., Bahdanau D., Bougares F., Schwenk H., Bengio Y. Learning Phrase Representations Using RNN Encoder-Decoder for Statistical Machine Translation. Proceedings of the 2014 Conference on Empirical Methods in Natural Language Processing (EMNLP).

[20] Luong M., Pham H., Manning C.D. Effective Approaches to Attention-based Neural Machine Translation. Proceedings of the 2015 Conference on Empirical Methods in Natural Language Processing (EMNLP).

[21] Woo S., Park J., Lee J.Y., Kweon I.S. CBAM: Convolutional block attention module. Proceedings of the European Conference on Computer Vision (ECCV).

[22] Fabius O., van Amersfoort J.R. Variational recurrent auto-encoders. Proceedings of the 3rd International Conference on Learning Representations, ICLR 2015-Workshop Track Proceedings.

[23] Sutskever I., Vinyals O., Le Q.V. Sequence to sequence learning with neural networks. Proceedings of the Advances in Neural Information Processing Systems.

[24] Shin H.K., Lee W., Yun J.H., Kim H.C. HAI 1.0: HIL-based augmented ICS security dataset. Proceedings of the CSET 2020—13th USENIX Workshop on Cyber Security Experimentation and Test, Co-Located with USENIX Security.

[25] Yoo K.M., Shin Y., Lee S.G. (2019). Data augmentation for spoken language understanding via joint variational generation. Proc. AAAI Conf. Artif. Intell..

[26] Xia C., Xiong C., Yu P., Socher R. Composed variational natural language generation for few-shot intents. Proceedings of the Findings of the Association for Computational Linguistics: EMNLP 2020.

[27] Arroyo D.M., Postels J., Tombari F. Variational transformer networks for layout generation. Proceedings of the IEEE Computer Society Conference on Computer Vision and Pattern Recognition.

[28] Zhang C., Kuppannagari S.R., Kannan R., Prasanna V.K. Generative Adversarial Network for Synthetic Time Series Data Generation in Smart Grids. Proceedings of the 2018 IEEE International Conference on Communications, Control, and Computing Technologies for Smart Grids, SmartGridComm.

[29] Smith K., Smith A.O. (2020). Conditional GAN for Timeseries Generation. arXiv.

[30] Marcheggiani D., Perez-Beltrachini L. Deep graph convolutional encoders for structured data to text generation. Proceedings of the 11th International Natural Language Generation Conference.

[31] Kipf T., Fetaya E., Wang K.C., Welling M., Zemel R. Neural relational inference for Interacting systems. Proceedings of the 35th International Conference on Machine Learning.

[32] Yu B., Yin H., Zhu Z. Spatio-temporal graph convolutional networks: A deep learning framework for traffic forecasting. Proceedings of the IJCAI International Joint Conference on Artificial Intelligence.

[33] Chung J., Kastner K., Dinh L., Goel K., Courville A., Bengio Y. A recurrent latent variable model for sequential data. Proceedings of the Advances in Neural Information Processing Systems.

[34] Ba J.L., Kiros J.R., Hiton G.E. (2016). Layer Normalization. arXiv.

[35] Kingma D.P., Ba J.L. Adam: A method for stochastic optimization. Proceedings of the 3rd International Conference on Learning Representations, ICLR 2015-Conference Track Proceedings.

[36] Sakoe H., Chiba S. (1978). Dynamic programming algorithm optimization for spoken word recognition. IEEE Transactions on Acoustics, Speech, and Signal Processing.

[37] van der Maaten L., Hinton G. (2008). Visualizing data using t-SNE. J. Mach. Learn. Res..

